# Inequalities in Ambulatory Care Sensitive Conditions: An International Comparison of High-Income Countries

**DOI:** 10.23889/ijpds.v9i5.2796

**Published:** 2024-09-18

**Authors:** Grace Spiro, Yu Qing Bai, ICCONIC Research Collaborative, Walter Wodchis

**Affiliations:** 1 University of Toronto

## Objective

To examine the differences in inequalities in Ambulatory Care Sensitive Conditions (ACSC) hospitalizations relative to socioeconomic status across nine countries, namely Australia, Canada, England, Finland, France, New Zealand, Spain, Switzerland, and the United States (US).

## Approach

The International Collaborative on Costs, Outcomes, and Needs in Care (ICCONIC) research collaborative developed national data sets with hospitalization and sociodemographic data aggregated to small-area levels and pooled across countries using a common data model. Inequalities were assessed using both the slope index of inequality and the relative index of inequality.

## Results

This study developed a common definition and set of codes for ACSC hospitalizations that proved to be comparable across countries and regions. Consistent socioeconomic gradients were observed in all countries with higher ACSC hospitalization rates for individuals in most disadvantaged areas. The greatest difference in hospitalizations between the highest and lowest income quintiles were observed in New Zealand (1603 per 100,000), Finland (1802 per 100,000) and England (1955 per 100,000).

## Conclusions

The high-income countries included in this study displayed many commonalities in inequalities in ACSC hospitalizations. As a proxy measure of primary care access and quality, the observed inequalities may implicate disparities in health care quality and access for the lowest socioeconomic status groups.

## Implications

Barriers to health care access in low socioeconomic groups remain a complex and multifaceted issue. Future research should investigate the underlying contributing factors to the observed ACSC hospitalization equity gradients, including primary care delivery model, remuneration approach, and population structure.

